# The evolving role of the dynamic thermal analysis in the early detection of breast cancer

**DOI:** 10.1186/1477-7800-2-8

**Published:** 2005-04-08

**Authors:** M Salhab, W Al Sarakbi, K Mokbel

**Affiliations:** 1 St George's and The Princess Grace Hospitals, London, UK

**Keywords:** Circadian rhythm, breast cancer, screening and dynamic thermal analysis

## Abstract

It is now recognised that the breast exhibits a circadian rhythm which reflects its physiology. There is increasing evidence that rhythms associated with malignant cells proliferation are largely non-circadian and that a circadian to ultradian shift may be a general correlation to neoplasia.

Cancer development appears to generate its own thermal signatures and the complexity of these signatures may be a reflection of its degree of development.

The limitations of mammography as a screening modality especially in young women with dense breasts necessitated the development of novel and more effective screening strategies with a high sensitivity and specificity. Dynamic thermal analysis of the breast is a safe, non invasive approach that seems to be sensitive for the early detection of breast cancer.

This article focuses on dynamic thermal analysis as an evolving method in breast cancer detection in pre-menopausal women with dense breast tissue. Prospective multi-centre trials are required to validate this promising modality in screening.

The issue of false positives require further investigation using molecular genetic markers of malignancy and novel techniques such as mammary ductoscopy.

## Introduction

Breast cancer is one of the most common cancers, it is estimated that one in eight women in the USA will develop breast cancer during their lifetime [1-4]. Furthermore, 25–30% of breast cancers are found in pre-menopausal women [1]. Currently mammography is the best available approach for the early detection of breast cancer in the general population with a sensitivity of 75–90% [2]. However, the positive predictive value is only 25% [3,4].

In addition to mammography, non invasive new modalities have been developed to allow the early detection of breast cancer in all age groups and more importantly in young women with dense breast tissue and women who have high risk of developing breast cancer such as, women with strong family history and carriers of BRCA1 and/or BRCA2 genes.

Currently, magnetic resonance imaging (MRI) is being studied for the early detection of breast cancer. Its sensitivity in high risk women has been found to be much higher than mammography but with a lower specificity [5,6]. Kriege et al observed a higher sensitivity for MRI in detection of breast cancer in women with a genetic predisposition or at high risk compared to (71% vs. 41 %) but with lower specificity (90% vs. 95%) [6].

Electrical impedance scanning (EIS) is another modality under development for breast cancer detection especially in young women with dense breasts [7]. The basic science behind its use is the fact that malignant tumours have lower electrical impedance than the surrounding normal tissue. However, separation between malignant and benign lesions needs further investigations [8].

Furthermore, mammary ductoscopy (MD) and visualization of mammary ducts and proteomics of nipple aspirate fluid (NAF) and serum are promising screening modalities that require further evaluation [9,10].

The limitations of mammography as a screening modality especially in young women with dense breasts necessitated the development of novel and more effective screening strategies with a high sensitivity and specificity.

This article focuses on the dynamic thermal analysis as an evolving non invasive and a safe method in breast cancer detection in pre-menopausal women with dense breast tissue and women at high risk due to family history or genetic predisposition.

## Breast and circadian rhythm [1]

It is now recognised that the establishment and growth of a tumour depend on neovascularization. This successful recruitment of new blood vessels into a tumour; also known as angiogenesis is dependent on angiogenic growth factors produced by the tumour cells [11]. Such new vessels grow adjacent to the tumour presumably to increase its nutrient supply [12]. These new vessels lack smooth muscles rendering them unreceptive to control by epinephrine [13,14]. The lack of receptivity produce a more constant blood flow, thus increasing the local temperature.

Earlier technology for assessing thermal abnormalities in the breast focussed on the presence of the abnormal temperature as a crucial marker [15-17]. In a study conducted by Gantherine et al, 21.3% of patients who had abnormal thermograms but no abnormality on physical examination and mammography developed breast cancer within the next 3 years [17]. In another study of women who had thermal abnormalities on initial examination using infrared technology, long term follow up (2–10 years) revealed that 33% of these women developed breast cancer, a rate six times higher than that expected in the normal population [18]. This relationship between breast skin temperature and breast cancer was thoroughly examined by Gros et al [15,16]. They found that the differences between the characteristics of rhythmic changes in skin temperature of clinically healthy and cancerous breasts were real and measurable. Despite these interesting observations thermography as a general screening tool for the detection of women at risk of breast cancer did not find a wide spread acceptance due to low sensitivity of the test and the subjective nature of the test interpretations.

The superficial thermal patterns measured on the surface of the breast seem to be related to tissue metabolism and vascularization within the underlying tissue. Such thermal patterns change significantly as a result of normal phenomena including menstrual cycle, pregnancy and more importantly the pathologic process itself. Additionally, cancer development represents the summation of a large number of mutations that occur over years, each with its own particular histologic phenotype [19-23].

Such changes appear to generate their own thermal signature and the complexity of these signatures may be a reflection of their degree of development [24-28].

Temperature in a normal breast increases from the skin into the deep tissue and heat conductivity in the healthy breasts is constant in most cases and generally can be characterized in terms of circadian rhythm periodicity [29]. In contrast, the rhythms associated with malignant cells proliferation are largely non circadian and suggest that a circadian to ultradian shift may be a general correlation to neoplasia. Heat production by the tumour under the influence of angiogenesis should be therefore re-examined in terms of absence of normal circadian fluctuations. Due to the increased blood flow and the lack of receptivity in the newly formed vessels in malignancy, temperature production exhibits circadian rhythmic variations to a far lesser degree than is evident in the healthy breasts [13]. It has been found that independent of a tumour's size, relatively small tumours (>/= 0.5 cm in diameter), poorly vascularized rapidly growing tumors can produce increases in regional heat. The explanation for this effect is unclear but it may be due to the chronic inflammatory response around developing breast tumours. With increasing evidence that inflammation can enhance tumor growth and is associated with a poor prognosis, this suggestion implies that thermal analysis may have considerable value [30].

Furthermore, the unique relationship between the thermal circadian rhythm and mitotic activity could be considered as a first warning of tumour development, which can be detected using a safe and non-invasive technology. The genes that drive the circadian rhythm are emerging as central players in gene regulation throughout the organism, particularly for cell-cycle regulatory genes and the genes of apoptosis [31].

## Dynamic thermal analysis

Recent technological advances have facilitated the recording of circadian rhythm variations of the breast and analysing the recorded data using highly complicated computer statistical software. A miniaturized microprocessor has been developed to record and store thermal information collected from eight separate sites of each breast. Sensors are placed in anatomically critical positions elicited by data obtained from tumour registries as to where cancers are most likely to develop.

In the First Warning System (FWS, Lifeline Biotechnologies, Florida, USA), thermal data are collected every five minutes for a period of 48 hours during which time women are encouraged to maintain their daily activities. 9000 pieces of data are recorded by microprocessors during the test period and analysed using specially developed statistical software. Temperature points from each contralateral sensor are plotted against each other to form a thermal motion picture of a lesion's physiological activity.

**Figure 1:**
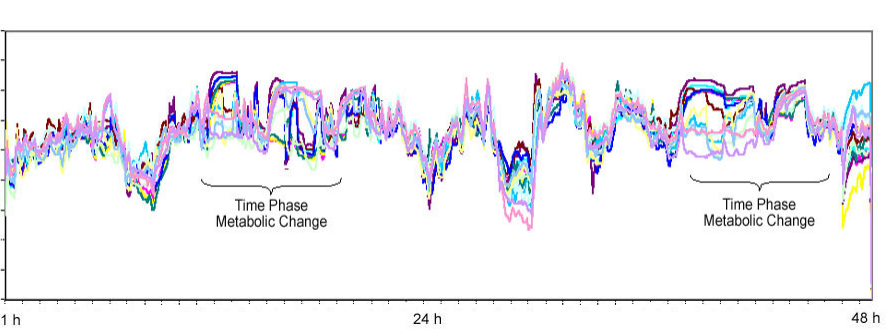
Dynamic thermal analysis in a patient with fibroadenoma.

**Figure 2:**
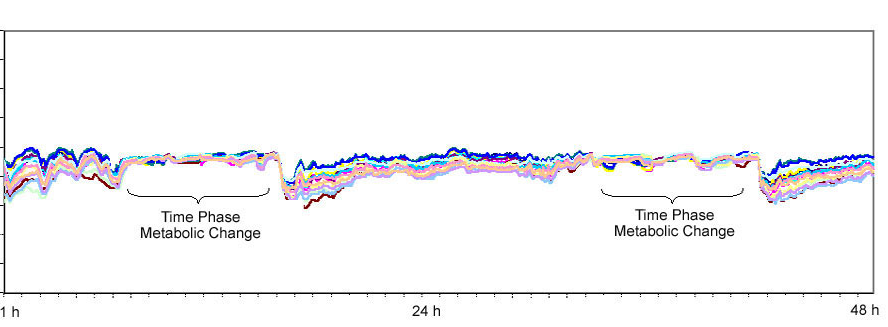
Dynamic thermal analysis in a patient with T1 breast cancer.

Such a technology was first used by Farrar et al who examined a cohort of 138 women who had been scheduled for open breast biopsies based on the finding of physical examination and mammography [14]. A total of 23 women (17%) were found to have breast cancer, of these, 20 (87%) were characterized by the monitor as being high risk. The other 3 patients (13%) who were missed by the monitor had ductal carcinoma. Mammography was positive or suspicious in only 19 patients (83%). Of the 4 cancers missed by mammography (3 of them were pre menopausal), the monitor correctly characterized 3 women as being high risk. Figures 1 and 2 demonstrate the thermal graphs in two patients with a fibroadenoma (Fig. 1) and a T1 breast cancer (Fig. 2). A neural net algorithm was subsequently developed and evaluated by the authors because of its value in analysing the non-linear data such as these recorded by the breast's monitors. Using this neural net algorithm reduced the number of false positives (18% vs. 30%)) and improved sensitivity (91% vs. 87%).

One of the main challenges to this technology is the false positive cases; confusion could be created in these women who are characterized as being positive or high risk by dynamic thermal analysis in the absence of physical and mammographical signs. This group of women may or may not have cancer in its earliest stages. Further retrospective analysis of the thermal data using a refined neural net algorithm may increase the sensitivity and reduce the number of false positives. Also this group of patients may well benefit from the new advances in the nipple aspirate fluid analysis and proteomic profiling technologies. Research is currently ongoing on this subject and the initial results are promising [9].

## The Future

Dynamic thermal analysis of the breast is a safe, non invasive approach that seems to be sensitive for the early detection of breast cancer especially in young women where the conventional mammography is of limited value. Such a technology could become the initial breast screening test in pre-menopausal women and those who are classified as positive can then be selected for anatomical imaging with mammography, MRI and/or ultrasonography. Further refinement of the neural net algorithm is required in order to shorten the period of data recording and improve specificity. Prospective multi-centre trials are then required to validate these promising observations. The issue of false positives require further investigation using molecular genetic markers of malignancy and novel techniques such as mammary ductoscopy [10].

Finally, a better understanding of the circadian rhythm biology [1,30] and clearer definition of the thermal activity boundaries for various pathological conditions of the breast will open the door to a new and more precise screening method for breast cancer.
